# Properties of Non-Structural Concrete Made with Mixed Recycled Aggregates and Low Cement Content

**DOI:** 10.3390/ma9020074

**Published:** 2016-01-26

**Authors:** Antonio López-Uceda, Jesús Ayuso, Martin López, José Ramón Jimenez, Francisco Agrela, María José Sierra

**Affiliations:** 1 Construction Engineering, University of Córdoba, Ed. Leonardo Da Vinci, Campus of Rabanales, Córdoba 14071, Spain; p62louca@uco.es (A.L.-U.); ir1loagm@uco.es (M.L.); ir1jiroj@uco.es (J.R.J.); fagrela@uco.es (F.A.); 2 Public Works Agency and Regional Ministry of Public Works and Housing of the Regional Government of Andalusia, Sevilla 41013, Spain; mjsierra@aopandalucia.es

**Keywords:** mixed recycled aggregate, ceramics, low cement content, non-structural concrete, mechanical properties, physical properties

## Abstract

In spite of not being legally accepted in most countries, mixed recycled aggregates (MRA) could be a suitable raw material for concrete manufacturing. The aims of this research were as follows: (i) to analyze the effect of the replacement ratio of natural coarse aggregates with MRA, the amount of ceramic particles in MRA, and the amount of cement, on the mechanical and physical properties of a non-structural concrete made with a low cement content; and (ii) to verify if it is possible to achieve a low-strength concrete that replaces a greater amount of natural aggregate with MRA and that has a low cement content. Two series of concrete mixes were manufactured using 180 and 200 kg/m^3^ of CEM II/A-V 42.5 R type Portland cement. Each series included seven concrete mixes: one with natural aggregates; two MRA with different ceramic particle contents; and one for each coarse aggregate replacement ratio (20%, 40%, and 100%). To study their properties, compressive and splitting tensile strength, modulus of elasticity, density, porosity, water penetration, and sorptivity, tests were performed. The results confirmed that the main factors affecting the properties analyzed in this research are the amount of cement and the replacement ratio; the two MRAs used in this work presented a similar influence on the properties. A non-structural, low-strength concrete (15 MPa) with an MRA replacement ratio of up to 100% for 200 kg/m^3^ of cement was obtained. This type of concrete could be applied in the construction of ditches, sidewalks, and other similar civil works.

## 1. Introduction

Most of the CO_2_-equivalent produced to manufacture concrete comes from cement production; over 400 kg of CO_2_-equivalent is generated per m^3^ of concrete [1,2], and is also responsible for 5% of all anthropogenic CO_2_ emissions [3]. In the European Union, 900 million tonnes of cement were produced in 2008 [4]. In the same year, CO_2_ equivalent emissions reached 101 million tonnes in the cement production sector [5]. This results in a high contribution to the emission of greenhouse gases, and contributes greatly to global warming.

Construction and demolition waste (CDW) represents almost a third of the total waste generated in the EU [5]. If CDW is not properly managed and is instead deposited in landfills, it can cause serious environmental problems, such as the release of contaminants that pollute surface and ground water [6,7]. Moreover, the recycling and reuse of CDW in new building materials require less energy consumption, reduce CO_2_ equivalent emissions, and, as a result, benefit the environment. Knoeri *et al.* [8] significantly reduced the environmental impact of recycled lean concrete by using 100% mixed rubble aggregates instead of conventional lean concrete. To promote the recycling and reuse of CDW, the Waste Framework Directive 2008/98/EC has mandated a 70% minimum CDW reuse and recycling rate by 2020. In Spain, the Second National Plan for CDW 2008–2015 [9] was developed to promote the recycling of this waste. This plan set a goal of achieving a recycling rate of 35% in 2015.

CDW consists of ceramic particles, mortar, concrete, and natural aggregates, as well as minor amounts of asphaltic material, gypsum, and impurities such as wood, metal particles, paper, and plastics. There are two major classifications of CDW aggregates, depending on their origin: recycled concrete aggregate (RCA), produced by crushing concrete, and mixed recycled aggregate (MRA), including at least 5% ceramic particles by weight. In Spain, RCA represents approximately 15%–20% and MRA approximately 80% of the total CDW aggregates produced [10].

In Spain, Structural Concrete Code EHE-08 [11] is the regulatory framework that sets the requirements for all materials used in concrete manufacturing, including recycled aggregates (RA). Among them, the fine fraction of RA is not allowed to be used in concrete manufacturing. The code only permits the use of the coarse fraction of RCA, which limits the replacement ratio of structural concrete to 20%. For non-structural concrete, coarse natural aggregates can be replaced by RCA up to 100%. Both cases exclude concretes manufactured using MRA. The standards in other countries, such as Germany, the United Kingdom, and Portugal, permit the partial or total use of MRA as the coarse fraction in non-structural concrete manufacturing, with different requirements in each country [12]. EHE-08 limits the minimum characteristic strength of non-structural concrete to 15 MPa and minimum cement content to 150 kg/m^3^. Because non-structural concretes are not steel reinforced, the EHE-08 code does not include any reference to the environment.

## 2. Literary Review

The possibility of using the coarse fraction of RCA for the partial or total replacement of the coarse fraction of natural aggregates (NA) in the manufacture of structural concrete has been studied by many researchers. It has been observed [13,14] that replacement ratios up to approximately 20% of RCA have marginal effects on the development of strength in concrete. Exteberría *et al.* [15] found that the strength of concrete made entirely with RCA was 20%–25% lower than conventional concrete after 28 days. Thomas *et al.* [13] found that a 20% replacement ratio led to minimal differences in water penetration under pressure and density values, approximately 5% lower than those of the control concrete. Malešev *et al.* [16] found a 44% increase in water absorption by sorptivity with total replacement using RCA with respect to the control concrete.

The effect of recycled aggregates made of pure ceramics has been explored by several authors, who used crushed bricks to replace the coarse fraction of NA. Their results are somewhat diverse. Brito *et al.* [17] measured strength losses of 45% with a replacement ratio of 100%, while Cachim [18] found up to 20% strength losses with 30% replacement. Similar results were found by Yang *et al.* [19], who observed a 10% reduction in strength with 20% replacement compared to the control concrete. Guerra *et al.* [20] obtained similar values when replacing up to 9% of the NA with recycled ceramic materials from sanitary porcelain debris.

However, a few studies have also been dedicated to the possibility of using MRA as a total or partial replacement material for the coarse fraction in the manufacture of concrete [21,22,23,24,25,26,27]. The objective of these studies was to obtain a structural concrete with a compressive strength greater than 25 MPa. The amount of cement used ranged from 240 kg/m^3^ to more than 300 kg/m^3^, so the amount of cement under 240 kg/m^3^ remains unexplored in studies with MRA incorporation in concrete. It was found that concrete made with MRA has a higher porosity, water absorption, and permeability and a lower strength than the control concretes that were made with NA and the same concrete mix composition.

The objectives of this work were as follows: (i) to analyze the mechanical and physical properties of a non-structural concrete made with MRA and a low cement content, and to study the effect of three factors: the replacement ratio of natural coarse aggregate by coarse MRA at four levels (0%, 20%, 40%, and 100%), the amount of ceramic particles in the MRA at two levels (14% and 30%) and the amount of cement at two levels (180 and 200 kg/m^3^ of concrete); and (ii) to verify if it is possible to achieve a low strength concrete that replaces a greater amount of natural aggregate with MRA that has a low cement content. This non-structural concrete could be applied to build ditches, floors, sidewalks, and paving blocks, for which a high mechanical strength is not necessary. The results of this research might have the double environmental benefit of reducing CO_2_ emissions by reducing the amount of cement and by recycling an RA of low quality, which represents the highest percentage of the CDW aggregates produced.

## 3. Materials and Experimental Details

### 3.1. Materials

#### 3.1.1. Mixed Recycled Aggregates

**Figure 1:**
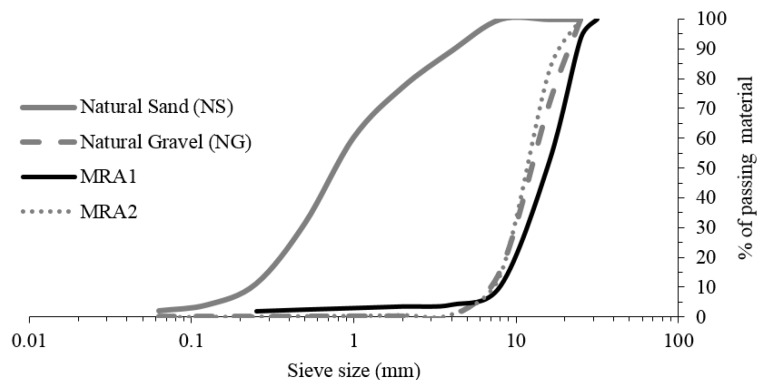
Particle size distribution of aggregates. Mixed recycled aggregates (MRA1 and MRA2).

Two mixed recycled aggregates (MRA1 and MRA2) with different percentages of ceramic particles collected on different days from a CDW treatment plant located in Córdoba (South of Spain) were used. These aggregates were by-products of the demolition of residential buildings. The grain size distribution of the materials is shown in Figure 1. Both materials were obtained by sieving the 0–25 mm fraction produced in the treatment plant.

Table 1 shows the physical and chemical properties as well as the main constituents of the recycled coarse aggregate with the RA constraints of the Spanish Code EHE-08. Only the water absorption requirement was not satisfied by MRA2, although, for RILEM (The International Union of Laboratories and Experts in Construction Materials, Systems and Structures) [28], the limit is less restrictive and up to 20% is allowed. It was noted that the amount of ceramic particles in MRA2 was greater than that in MRA1, so that, the absorption was higher in MRA2. MRA2 complies with the EHE-08 requirements, whereas MRA1 does not, as its total sulfur content slightly exceeds the EHE-08 limit. In the literature review, different classifications have been proposed based on properties or compositions of the two MRA. Agrela *et al.* [29] established a classification for RA that depends on the ceramic and concrete particle content. In this scheme, MRA1 was classified as mixed recycled aggregate (MixRA), because its ceramic content was between 10%–30% by weight. MRA2 was classified as a ceramic recycled aggregate (CerRA) because its ceramic content was over 30% by weight. Silva *et al.* [30] suggested a different RA classification based on the oven-dried density, water absorption, and Los Angeles (LA) abrasion value. In the latter scheme, MRA1 was classified as B-II because its oven-dried density was higher than 2.2 Mg·m^−3^, and its water absorption and LA values were lower than 6.5% and 45, respectively. Conversely, MRA2 was classified as C-I because its oven-dried density was over 2.0 Mg·m^−3^, and its water absorption and LA values were lower than 10.5% and 50, respectively.

**Table 1:** Physical, chemical properties and components of mixed recycled aggregates (MRA). SSD: Saturated surface dry.

**Physical Properties**	**According to Standard**	**MRA1**	**MRA2**	**EHE-08 Requirements**
Water absorption (%)	UNE-EN 1097-6:2014 [31]	6.1	9.0	<5% General, <7% RCA
Oven-dried density (Mg/m^3^)	UNE-EN 1097-6:2014 [31]	2.24	2.08	-
SSD density (Mg/m^3^)	UNE-EN 1097-6:2014 [31]	2.38	2.27	-
Flakiness index (%)	UNE-EN 933-3:2012 [32]	10.8	14.7	<35
Los Angeles Abrasion test	UNE-EN 1097-2:2010 [33]	35.6	32.3	<40
Freeze-thaw resistance (%)	UNE-EN 1367-2:2010 [34]	5.2	14.0	<18%
**Chemical properties**	**According to Standard**	**MRA1**	**MRA2**	**EHE-08 Requirements**
Total sulfur content (% S)	UNE-EN 1744-1-11:2010 [35]	1.02	0.96	<1
Acid-soluble sulfates (% SO_3_)	UNE-EN 1744-1-12:2010 [36]	0.65	0.62	<0.8
Chlorides (%)	UNE-EN 1744-1-7:2010 [37]	<0.01	<0.01	<0.05
Components (%)	UNE-EN 933-11:2009 [38]	-	-	-
Asphalt	-	0.9	0.5	-
Ceramics	-	13.9	30.2	-
Mortar and concrete	-	49.0	44.6	-
Unbound aggregates	-	34.9	24.0	-
Gypsum	-	0.4	0.5	-
Others (wood, glass, plastic, and metal)	-	0.9	0.2	-

#### 3.1.2. Natural Aggregates

Figure 1 illustrates the grain size distribution of natural siliceous sand (NS) with a maximum size of 4 mm, and siliceous gravel (NG) with a 6–25 mm fraction. The most important physical and chemical properties of natural aggregates for concrete production are summarized in Table 2.

**Table 2:** Physical and chemical properties of natural aggregates (NA). NS = Natural siliceous sand, NG = Siliceous gravel.

**Physical Properties**	**According to Standard**	**NS**	**NG**
Water absorption (%)	UNE-EN 1097-6:2014 [31]	0.92	0.73
SSD density (Mg/m^3^)	UNE-EN 1097-6:2014 [31]	2.66	2.70
Flakiness index (%)	UNE-EN 933-3:2012 [30]	-	20.60
Los Angeles abrasion test	UNE-EN 1097-2:2010 [33]	-	18.10
Friability test	UNE 83115:1989 [39]	12.40	-
**Chemical properties**	**According to Standard**	**NS**	**NG**
Total sulfur content (% S)	UNE-EN 1744-1-11:2010 [35]	0.36	0.57
Acid-soluble sulfates (% SO_3_)	UNE-EN 1744-1-12:2010 [36]	0.17	0.51
Chlorides (%)	UNE-EN 1744-1-7:2010 [37]	<0.01	<0.01

#### 3.1.3. Cement

A CEM II/A-V 42.5 R type Portland cement was used. The cement properties are shown in Table 3. The cement used for this study had a fly ash content of 17%, which was produced from the emissions of a nearby coal-fired power plant. This represents a significant benefit in CO_2_ emission reduction [40].

**Table 3:** Chemical composition and physical properties of cement.

Loss on Ignition (%)	Specific Mass (Mg/m^3^)	Blaine Specific Surface Area (m^2^/kg)	SiO_2_	Al_2_O_3_	Fe_2_O_3_	MgO	CaO	Na_2_O	K_2_O
			(%)						
1.38	2.89	351.9	26.49	8.70	3.31	1.41	54.36	3.26	1.43

#### 3.1.4. Admixtures

Two admixtures were used in this study. The plasticizer Conplast MR260 is formulated as a mixture of synthetic and natural polymers. Its main function is to increase the workability of a material. The superplasticizer Conplast SP420 is based on organic polymers. Its main function is to reduce the water-to-cement ratio.

### 3.2. Experimental Details.

#### 3.2.1. Mix Proportions

The mix proportion was a commercial design provided by PREBESUR SL (Córdoba, Spain), an industrial concrete plant located in Córdoba (Spain). The concrete mixes were designed to evaluate the influence of the following factors on the mechanical and durability properties of concrete:

- Amount of cement. Two cement contents were used: 180 and 200 kg/m^3^.
- Replacement ratio of coarse aggregate. Four levels were used: 0%, 20%, 40%, and 100%. The replacement percentage was calculated using the equivalent volume.
- Type of MRA. Two MRA (MRA1 and MRA2) were tested, with different percentages of ceramic particles.

Two series of concrete mixes were produced with a constant water-to-cement ratio of 0.65: one for a cement content of 180 kg/m^3^ and the other for a cement content of 200 kg/m^3^. Each series consisted of seven concrete mixes: one with natural aggregates that acted as control concrete (CC); one for each type of MRA (CMRA1 and CMRA2); and one for each replacement ratio (20%, 40%, and 100%). Table 4 and Table 5 illustrate the concrete mix proportions for each series.

**Table 4:** Composition of the concrete mixes for Series I (180 kg of cement/m^3^). CC: Control concrete.

Samples	Replacement Ratio (%)	Proportions (kg/m^3^)						
		Cement	Water	NS	NG	MRA	Plasticizer	Superplasticizer
CC-I	0	180	117	1100	950	0	1.92	2.15
CMRA1-20-I	20	180	117	1100	759	147	1.92	2.15
CMRA1-40-I	40	180	117	1100	569	294	1.92	2.15
CMRA1-100-I	100	180	117	1100	0	735	1.92	2.15
CMRA2-20-I	20	180	117	1100	817	144	1.92	2.15
CMRA2-40-I	40	180	117	1100	613	288	1.92	2.15
CMRA2-100-I	100	180	117	1100	0	720	1.92	2.15

**Table 5:** Composition of the concrete mixes for Series II (200 kg of cement/m^3^).

Samples	Replacement Ratio (%)	Proportions (kg/m^3^)						
		Cement	Water	NS	NG	MRA	Plasticizer	Superplasticizer
CC-II	0	200	130	1070	950	0	2.13	2.39
CMRA1-20-II	20	200	130	1070	759	147	2.13	2.39
CMRA1-40-II	40	200	130	1070	569	294	2.13	2.39
CMRA1-100-II	100	200	130	1070	0	735	2.13	2.39
CMRA2-20-II	20	200	130	1070	817	144	2.13	2.39
CMRA2-40-II	40	200	130	1070	613	288	2.13	2.39
CMRA2-100-II	100	200	130	1070	0	720	2.13	2.39

To increase the workability and reduce the amount of water, two additives were used in all the mixes: plasticizer, with a density of 1.184 g/cm^3^ and superplasticizer, with a density of 1.195 g/cm^3^, were added at 9 mL/kg and 10 mL/kg of cement, respectively. The target was to achieve an S3 slump class, according to UNE-EN-206-1:2008 [41].

#### 3.2.2. Mixing Process

**Figure 2:**
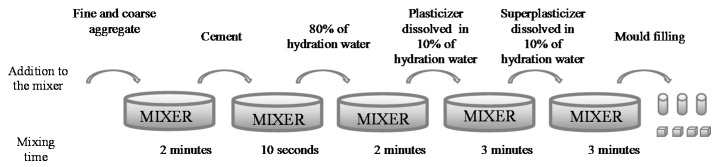
Mixing process diagram.

MRA have a high water absorption capacity that reduces the workability of fresh concrete and water available for cement hydration. Therefore, some authors [25,42] recommend an initial wetting of the MRA before the mixing process. As such, the MRA were flooded for 10 minutes prior to mixing. It was estimated that during this wetting period MRA absorb 80% of their total capacity [24]. Figure 2 presents the scheme of the mixing process.

#### 3.2.3. Test Method

The mechanical and physical properties were measured at an age of 28 days. Three samples were tested for each test. All the specimens were demoulded at 24 h and then cured in a chamber at constant temperature (23 °C ± 2 °C) and relative humidity (95% ± 5%). Table 6 summarizes all the tests that were performed.

**Table 6:** Tests performed to study the properties of concrete.

Test	Standards	Form and Sample Dimensions
Slump test for workability	UNE-EN 12350-2:2009 [43]	Cubic: 100 × 100 × 100 mm
Compressive strength	UNE-EN 12390-3:2009 [44]	Cylindrical: Ø 150 × 300 mm
Tensile splitting strength	UNE-EN 12390-6:2009 [45]	Cylindrical: Ø 100 × 200 mm
Static modulus of elasticity in compression	UNE 83316:1996 [46]	Cylindrical: Ø 150 × 300 mm
Density of hardened concrete	UNE-EN 12390-7:2009 [47]	Cubic: 150 × 150 × 150 mm
Porosity of hardened concrete	UNE-EN 12390-7:2009 [47]	Cubic: 150 × 150 × 150 mm
Penetration of water under pressure	UNE-EN 12390-8:2009 [48]	Cylindrical: Ø 150 × 300 mm
Determination of sorptivity	UNE- EN 1925:1999 [49]	Cubic: 100 × 100 × 100 mm

## 4. Results and Discussion

The mean values of the results of all tests carried out for each concrete mix with the coefficients of variation are shown in Table 7. All the coefficients of variation were low, which justifies the use of only three replicates. To assess the significance of the effect of the three categorical factors on each of the properties, an analysis of variance (ANOVA) was conducted with the statistical software Statgraphics Centurion XVI (Version 16.1.18, StatPoint Technologies, Inc., Warrenton, VA, USA). The *F*-test in the ANOVA analysis was used to evaluate whether one factor had statistically significant effects on the properties studied, with a 95% confidence level. If the *p*-value was lower than 0.05, the factor showed a significant effect on the property studied. To check whether there was a significant difference between the groups for each factor, Fisher’s Least Significant Difference (LSD) test was conducted to examine the mean plot and identify the LSD intervals that did not overlap.

Table 8 shows a summary of the results obtained with the ANOVA. The results indicate that the percentage of replacement has an influence on all the mechanics and physical properties analyzed and the type of aggregate has no influence on any of the properties, which proves that the two recycled aggregates used in this research were of comparable characteristics. The amount of cement has influence on compressive and splitting tensile strength, as well as on the density and penetration of water under pressure. Additionally, the degrees of freedom n1 and n2 (n1 is equal to factor levels minus one and n2 is equal to the number of observations minus factor levels) considered for the *F*-Snedecor contrast are indicated.

**Table 7:** Means values of the results of all tests.

Samples	*f*_ccub,_ MPa	*c.v.*	*f*_ccyl,_ MPa	*c.v.*	Splitting Tensile Strength, MPa	*c.v.*	Modulus of Elasticity, GPa	*c.v.*	SSD-Density, Mg·m^−3^	*c.v.*	Porosity, %	*c.v.*	Water Penetration, mm	*c.v.*	Sorptivity, mm·h^−1/2^	*c.v.*
CC-I	29.0	1.37	20.8	2.53	2.42	2.74	15.3	3.94	2.31	0.74	11.7	1.41	57.0	6.24	0.57	7.17
CMRA1-20-I	23.8	1.01	19.6	1.68	2.36	3.78	14.5	2.35	2.29	0.50	11.8	2.16	89.0	3.31	0.59	6.92
CMRA1-40-I	20.5	4.26	18.6	0.90	2.02	3.52	12.5	5.94	2.25	0.44	13.3	1.62	96.0	3.90	0.76	6.45
CMRA1-100-I	18.5	1.14	17.3	4.86	1.58	15.68	10.5	9.06	2.24	0.10	14.1	0.72	97.5	7.26	0.94	4.84
CMRA2-20-I	21.7	1.95	20.0	1.45	2.35	6.02	14.4	2.96	2.26	0.19	12.2	1.39	63.0	9.07	0.62	12.58
CMRA2-40-I	21.1	0.56	19.2	0.21	2.10	2.26	12.8	3.58	2.24	0.42	13.1	1.27	74.7	7.91	0.81	9.07
CMRA2-100-I	20.5	0.82	19.0	0.77	1.97	10.58	11. 9	1.56	2.20	0.47	13.9	0.37	76.5	3.74	0.99	7.05
CC-II	34.6	2.74	25.8	0.54	2.81	1.28	17.6	3.87	2.38	0.51	10.9	1.35	34.0	8.66	0.25	14.93
CMRA1-20-II	32.7	1.20	24.8	1.43	2.60	3.02	15.8	2.84	2.36	0.23	11.3	1.10	45.3	9.24	0.36	9.46
CMRA1-40-II	30.1	0.42	23.6	0.97	2.29	3.97	15.5	2.86	2.33	0.31	13.2	0.88	52.7	5.44	0.55	3.93
CMRA1-100-II	22.8	4.26	20.5	0.90	2.18	3.52	11.6	8.60	2.25	0.54	13.9	1.43	78.0	6.28	0.89	6.66
CMRA2-20-II	34.5	0.63	25.0	1.77	2.63	2.19	16.7	2.29	2.30	0.62	11.5	1.97	36.3	7.16	0.56	5.15
CMRA2-40-II	33.8	3.66	24.5	1.29	2.35	2.83	16.2	2.47	2.29	0.16	13.1	0.94	45.3	7.28	0.73	5.59
CMRA2-100-II	27.6	3.94	23.4	1.20	2.21	7.23	13.1	4.83	2.25	0.50	13.5	0.10	76.5	6.94	1.03	3.76

*f*_ccub_ = Compressive strength in cubic specimens; *f*_ccyl_ = Compressive strength in cylindrical specimens; *c.v.* = Coefficient of variation (%).

**Table 8:** Summary results of ANOVA and coefficient of variation.

Properties		Factors							
		Amount of Cement (kg/m^3^)		(%) of Replacement				Type or MRA	
-	Factor levels	180	200	0	20	40	100	1	2
	Degrees of freedom	(1;12)		(3;10)				(1;10)	
Compressive strength (*f*_ccyl_)	*p*-value	<0.0001		<0.0001				0.5477	
	*c.v.*	5.3	6.7	7.4	3.6	2.1	9.9	8.1	2.9
Tensile splitting strength	*p*-value	0.0439		<0.0001				0.6006	
	*c.v.*	13.0	9.2	13	8.5	4.5	16.4	12.9	7.5
Modulus of elasticity in compression	*p*-value	0.0647		<0.0001				0.5225	
	*c.v.*	11.9	12.9	13.9	8.0	4.4	21.6	14.5	9.5
Density of hardened concrete	*p*-value	0.0443		<0.0001				0.2568	
	*c.v.*	1.5	2.0	2.7	1.4	0.7	2.3	1.6	1.5
Porosity of hardened concrete	*p*-value	0.5132		<0.0001				0.9602	
	*c.v.*	7.1	9.0	12.3	8.5	4.0	8.5	8.2	6.3
Penetration of water under pressure	*p*-value	0.0023		0.0016				0.1081	
	*c.v.*	18.5	31.6	44.9	22.3	14.1	24.1	20.1	22.6
Sorptivity	*p*-value	0.3218		<0.0001				0.3875	
	*c.v.*	20.8	41.3	72.0	32.4	9.9	29.6	27.3	26.2

*c.v.* = Coefficient of variation (%); bold *p*-values show significant difference.

### 4.1. Effect of Cement Content

#### 4.1.1. Mechanical Properties

**Figure 3:**
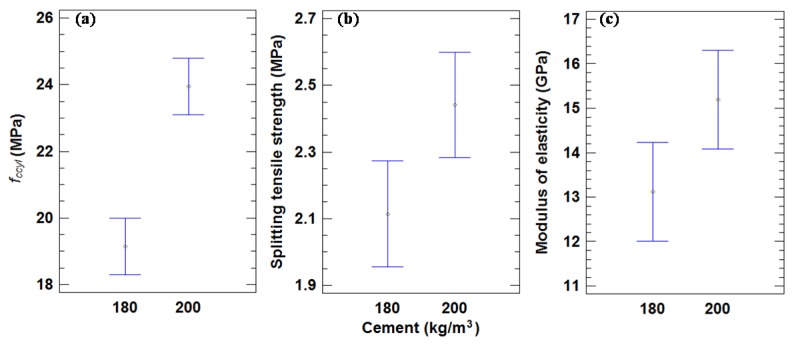
(**a**) Mean values of compressive strength in cylindrical specimens and 95% LSD intervals *vs.* amount of cement. (**b**) Mean values of splitting tensile strength and 95% LSD intervals *vs.* amount of cement. (**c**) Mean values of modulus of elasticity 95% LSD intervals *vs.* amount of cement.

As seen in Figure 3, the mean values for all of the mixes of cylindrical specimens (*f*_ccyl_ values) with 200 and 180 kg of cement/m^3^ were 24.0 and 19.2 MPa, respectively, with a decrease of 19.8%. There were statistically significant differences at a 95% confidence level, as is indicated by the non-overlapping bars in Figure 3. Mas *et al.* [23] obtained a greater compressive strength decrease of 46.4% between 360 and 240 kg of cement/m^3^ series, due to a higher cement content.

According to the ACI Code 318-08 [50], the mean compressive strength value (*f*_cm_) at 28 days for concrete with a characteristic compressive strength (*f*_ck_) under 21 MPa, when there are insufficient data to establish a standard deviation of the sample, is given by the following expression:

*f*_cm_ = *f*_ck_ + 7 (1)

The values of *f*_ck_ estimated by equation [1] for each concrete mixture are shown in Table 9. Six of the mixes, corresponding to a cement content of 200 kg/m^3^, had an *f*_ck_ greater than 15 MPa, which complies with the requirements of the Spanish standard EHE-08 for non-structural concrete. Only the concrete made with 100% MRA1 replacement had a slightly lower *f*_ck_ value (14 MPa). None of the other mixes (180 kg/m^3^) complied with the EHE-08 requirement. This fact does not mean that they cannot be used in applications such as drainage ditches, sidewalks, trench filling and other non-structural uses, whose strength requirements are very low.

**Table 9:** The *f*_ck_ values estimated by ACI Code 318-08.

**Cement content**	**180 kg of Cement/m^3^**						
**Samples**	**CC**	**CMRA1-20**	**CMRA1-40**	**CMRA1-100**	**CMRA2-20**	**CMRA2-40**	**CMRA2-100**
*f*_cm_	21	20	19	17	20	19	19
*f*_ck_	14	13	12	10	13	12	12
**Cement content**	**200 kg of Cement/m^3^**						
**Samples**	**CC**	**CMRA1-20**	**CMRA1-40**	**CMRA1-100**	**CMRA2-20**	**CMRA2-40**	**CMRA2-100**
*f*_cm_	26	25	24	21	25	25	23
*f*_ck_	19	18	17	14	18	18	16

**Figure 4:**
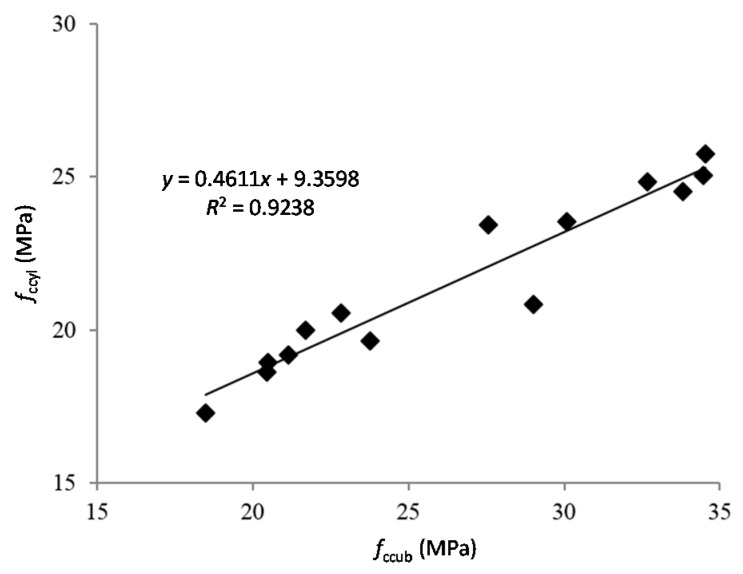
Correlation between compressive strength values for cylinder and cubic specimens at 28 days.

The relationship between the compressive strengths of cylindrical and cubic specimens is plotted in Figure 4, with a good linear relationship (*R*^2^ = 0.92).

Table 7 indicates the mean splitting tensile strength values for each concrete mixture. The non-overlapping bars in Figure 3 show that there were statistically significant differences in the splitting tensile strength based on the amount of cement with a 95% confidence level. The mean values of the splitting tensile strength for all of the mixes with 200 and 180 kg of cement/m^3^ were 2.44 and 2.12 MPa, respectively, with a decrease of 13.3% from the higher content to the lower one. Mas *et al.* [23] obtained a higher splitting tensile strength decrease (35.9%). This agrees with the statement of Neville [51] who affirmed that the compressive and tensile strengths decline with the cement content, but the latter at a lower rate.

The mean values of the static modulus of elasticity are shown in Table 7. The mean values for all of the mixes with 200 and 180 kg of cement/m^3^ were 15.2 and 13.1 GPa, respectively. The decrease in the modulus of elasticity between both mixes was 13.8%, which is similar to the splitting tensile strength decrease (13.3%). Figure 3 shows that there were no statistically significant differences between the two cement contents in modulus of elasticity property, as indicated by the overlapping bars.

#### 4.1.2. Physical Properties

**Figure 5:**
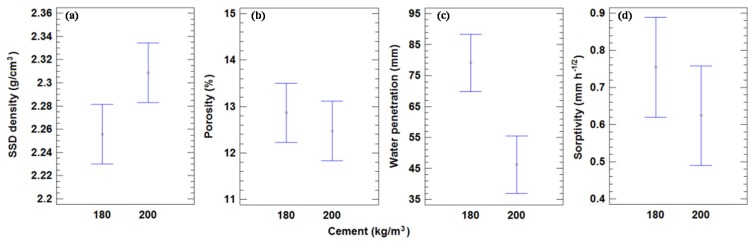
(**a**) Mean values of SSD density and 95% LSD intervals *vs.* amount of cement. (**b**) Mean values of porosity and 95% LSD intervals *vs.* amount of cement. (**c**) Mean values of water penetration and 95% LSD intervals *vs.* amount of cement. (**d**) Mean values of sorptivity and 95% LSD intervals *vs.* amount of cement.

Four different physical properties of concrete, namely the saturated surface dry density (SSD density), water penetration under pressure, porosity, and water sorptivity were estimated. The results are shown in Table 7. The mean density values for all of the mixes with 200 and 180 kg of cement/m^3^ were 2.31 and 2.26 Mg/m^3^, respectively. The decrease in the SSD density between the two series was 2.25%. In contrast, Mas *et al.* [23] obtained a decrease of 1.28% between 360 and 240 kg of cement/m^3^. The analysis shown in Figure 5 did not yield any statistically significant differences at a 95% confidence level, indicated by the lack of overlapping bars.

The mean values of the porosity are given in Table 7. Figure 5 indicates that a higher cement content reduces the porosity and consequently increases the density. There were no significant differences in porosity with a 95% confidence level. The mean values for all mixes with 200 and 180 kg of cement/m^3^ were 12.5% and 12.9%, respectively, with an increase of 3.2% between both series.

The mean values of water penetration under pressure for all concrete mixtures are given in Table 7, and the value for the mixes with 200 and 180 kg of cement/m^3^ were 52.6 and 79.1 mm, respectively, (a difference of 50.4%). This is consistent with the higher porosity and lower density of the second series. Figure 5 shows that there were significant differences between the series, as the amount of cement had a great effect on the water penetration under pressure. The results of Mas *et al.* [23] showed an increase of 114% between 360 and 240 kg of cement/m^3^, which is greater than that seen in our results. This could be attributed to the lower cement content (10%) in the present probes than in those used in their tests (33%).

The mean values of sorptivity for all the mixes with 200 and 180 kg of cement/m^3^ were 0.62 and 0.75 mm·h^−1/2^, respectively, with an increase of 20.2%.

**Figure 6:**
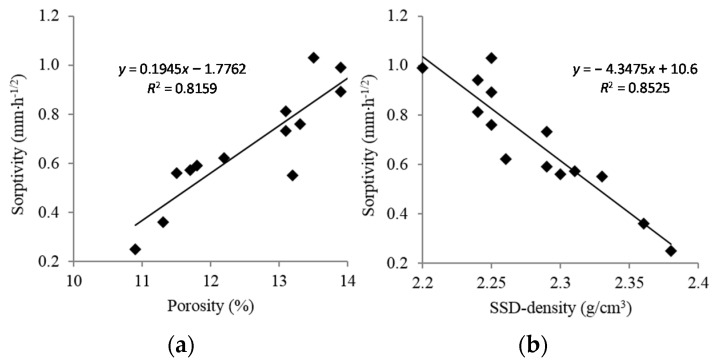
(**a**) Correlation between porosity with sorptivity. (**b**) Correlation between SSD density with sorptivity.

Strong linear relationships were found between the sorptivity, density, and porosity, as depicted in Figure 6. Sorptivity declines as the density increases and increases as the porosity increases.

### 4.2. Effect of Replacement Ratio

#### 4.2.1. Mechanical Properties

**Figure 7:**
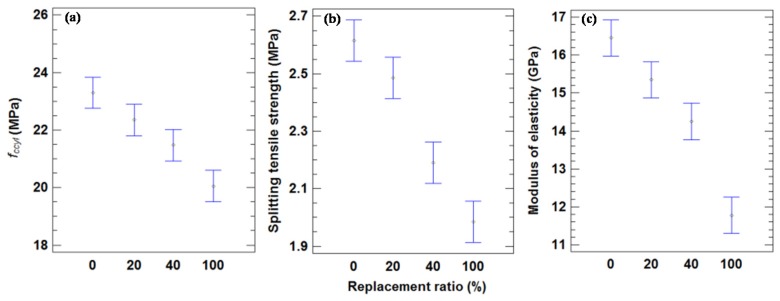
(**a**) Mean values of compressive strength in cylindrical specimens and 95% LSD intervals *vs.* replacement ratio. (**b**) Mean values of splitting tensile strength compressive strength in cylindrical specimens and 95% LSD intervals *vs.* replacement ratio. (**c**) Mean values of modulus of elasticity and 95% LSD intervals *vs.* replacement ratio.

The mean compressive strength of the concrete mixes with 20% of the coarse aggregate replaced was 4% less than the mean value of the control mixes after 28 days. The non-overlapping bars in Figure 7 indicate that there was no statistically significant difference between these series of mixes. Mas *et al.* [25] concluded that the reduction in the mean compressive strength after 28 days in concrete made with coarse MRA was 8.1% for a 20% replacement level and 250 kg of cement/m^3^. This could be attributed to the fact that in a concrete manufactured with low cement content, the quality of coarse aggregate has a minor influence on strength.

The result was 7.8% less than the mean value of CCs with a 40% replacement ratio, and statistically significant differences in these two series of mixes did occur. These results contrast with those of Medina *et al.* [24], who found an 18.4% difference with 323 kg of cement/m^3^ and 50% replacement ratio. Their result could be attributed to the larger amount of cement used.

In the case of the total replacement of coarse aggregate, the decrease was 13.9%, significantly different from the other mixes. Martinez-Lage *et al.* [26] estimated the loss for concrete with 100% replacement was 23%, with values ranging from 20% for 250 kg of cement/m^3^ to 31% for 290 kg of cement/m^3^. These results were similar to those obtained by Ihobe [21], who found a 25% decrease relative to the control concrete with 250 kg of cement/m^3^; Brito *et al.* [17] reached a reduction of 43.48% for total replacement with 346.7 kg of cement/m^3^. These results indicate that for a percentage of replacement, the loss of strength was smaller as the amount of cement decreased. This observation agrees with findings by Mas *et al.* [23], who found that concretes with MRA had lower percentages of reduction of unconfined compressive strength than concretes with a higher cement content.

**Figure 8:**
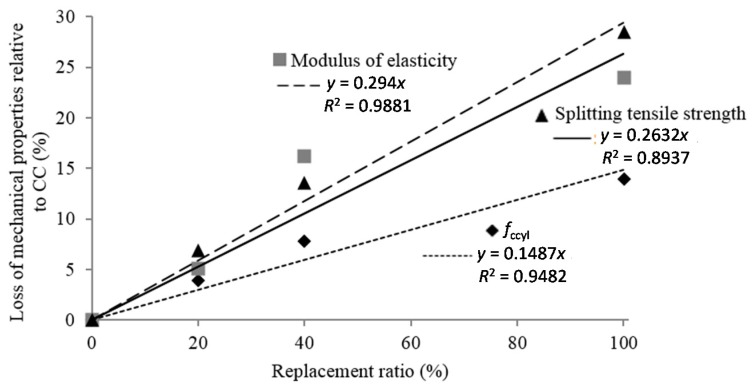
Loss of mean compressive strength, tensile strength, and modulus of elasticity relative to control concrete (CC) in relation to the replacement ratio.

Figure 8 represents the loss of mean values of the mechanical properties (*f*_ccyl_), splitting tensile strength and modulus of elasticity, with the replacement ratio. A strong linear relationship exists between the loss of mechanical properties relative to CCs and the replacement ratio.

No statistically significant differences in the splitting tensile strength (95% confidence level) between CCs and a 20% replacement level were observed in the data of Figure 7. The loss of strength was 5.0%; this result agrees with findings by Mas *et al.* [23], who measured 6.8% for the same replacement level with 240 kg/m^3^ of CEM II.

For 40% replacement, the decrease in splitting tensile strength relative to CCs was 16.2%, which is a statistically significant difference from both a 20% replacement level mix and the CCs. These results contrast with those of Mas *et al.* [25], who concluded that the reduction in average tensile strength was 10% for a 40% replacement level with 240 kg of cement/m^3^.

A 24% decrease was measured with total replacement with respect to CC. Yang *et al.* [19] obtained a 30.5% loss for concrete with 100% mixed recycled aggregate for 435 kg of cement/m^3^. This higher reduction is due to the larger cement content used in their study.

The modulus of elasticity varied in the same way as the compressive and splitting tensile strength. The mean value of the modulus of elasticity with a 20% replacement level was 15.34 GPa. This was 6.8% less than the mean value for CCs (16.46 GPa). For a 40% replacement level, the mean value was 14.22 GPa, with a decrease of 13.6% relative to CCs. With full replacement, the decrease was 28.4%. This result is in accordance with the results of Martinez-Lage *et al.* [26] and Ihobe [21], who estimated decreases of 34% and 28%, respectively, for total replacement in concrete manufactured with MRA. Figure 7 shows that there were statistically significant differences between all replacement ratios.

#### 4.2.2. Physical Properties

**Figure 9:**
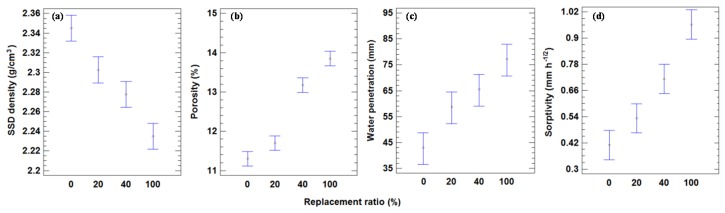
(**a**) Mean values of SSD density and 95% LSD intervals *vs.* replacement ratio. (**b**) Mean values of porosity and 95% LSD intervals *vs.* replacement ratio. (**c**) Mean values of water penetration and 95% LSD intervals *vs.* replacement ratio. (**d**) Mean values of sorptivity and 95% LSD intervals *vs.* replacement ratio.

Figure 9 shows that there were significant differences between the SSD density of CCs and all replacement ratios. The SSD density decreased as the replacement levels of the MRA increased; this was due to the low density of MRA compared to NA. The mean SSD density for 20%, 40%, and 100% replacement ratios, decreased by 1.8%, 2.9%, and 4.4%, respectively. Mas *et al.* [25] found a 3.3% loss of density for a concrete with 240 kg/m^3^ of CEM II and 20% MRA replacement. Martinez-Lage *et al.* [26] found a 7.7% decline with a 100% MRA replacement and 250–290 kg/m^3^ of CEM II. These higher values could be attributed to the larger cement content used.

As seen in Figure 9, there were significant differences in porosity between all replacement levels, showing that this factor had an important effect on porosity. The mean value of porosity for 20%, 40%, and 100% replacement levels increased relative to CCs by 3.4%, 16.3%, and 22.4%, respectively. Beltran *et al.* [52] found 1.75% and 6.3% growth for a concrete manufactured with 300 kg of cement/m^3^ and with a 20% and 100% replacement of coarse NA by RCA, respectively. This minor growth could be attributed to the greater density of RCA compared to MRA.

**Figure 10:**
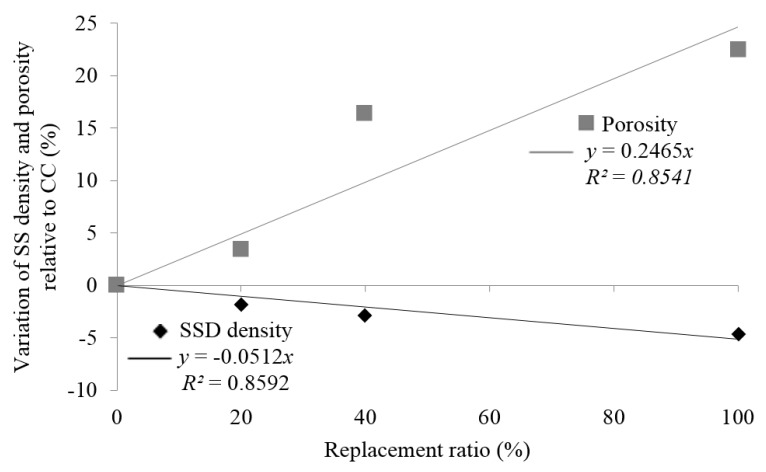
Loss of mean of SSD density and porosity variation relative to CC in relation to the replacement ratio.

Figure 10 indicates that a strong linear correlation exists between the decrease in SSD density (*R*^2^ = 0.86) and replacement ratio, and the increase in porosity (*R*^2^ = 0.85) and replacement ratio.

Figure 9 shows that there were no significant differences between CCs and a 20% replacement mix in water penetration under pressure. A 28.4% increment of maximum water penetration under pressure was found with a 20% replacement level relative to the control. Total replacement resulted in an 80.4% increment of maximum water penetration under pressure relative to CCs.

**Figure 11:**
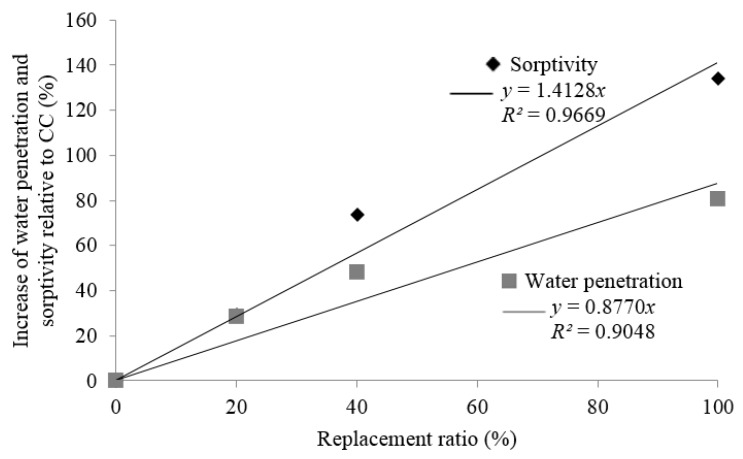
Loss of mean of water penetration and sorptivity increase relative to CC in relation to the replacement ratio.

Figure 11 indicates that the maximum water penetration under pressure and sorptivity increases linearly with the replacement ratio, with a high correlation index, 0.90 and 0.97, respectively. The maximum water penetration under pressure for the series with 200 kg of cement/m^3^ varied between 34 mm for CCs and 78 mm for 100% replacement with MRA1. These results agree with those obtained by Mas *et al.* [25] and Correia *et al.* [53].

Figure 9 does not show significant differences (95% confidence level) between CCs and 20% replacement in the sorptivity property. A 20% replacement ratio relative to CCs produced a 29.2% increase in sorptivity; this may be due to the higher porosity of concrete with 20% replacement.

For 40% replacement, the increase in sorptivity relative to CCs was 73.4%, similar to Etxeberría *et al.* [27] (65.2%) for similar probes (23% ceramic content of MRA and 240 kg of CEM II/A-V/m^3^). Medina *et al.* [24] found an increase of 13% for 50% substitution; this lower value could be attributed to the larger cement content used (323 kg of CEM I/m^3^), resulting in a higher density, as well as the lower ceramic material content (5.3%) in relation to that used in this study (13.9% for MRA1 and 30.2% for MRA2). For total replacement, the gain in sorptivity was 134.1% relative to CCs, which is very similar to the results obtained by Sanchez [22], who found a 133% increase with 240–265 kg of cement/m^3^. Conversely, Correia *et al.* [53] found an increase of 70.4%, but with a cement content of 346.7 kg/m^3^.

### 4.3. Effect of Type of Aggregate

#### 4.3.1. Mechanical Properties

**Figure 12:**
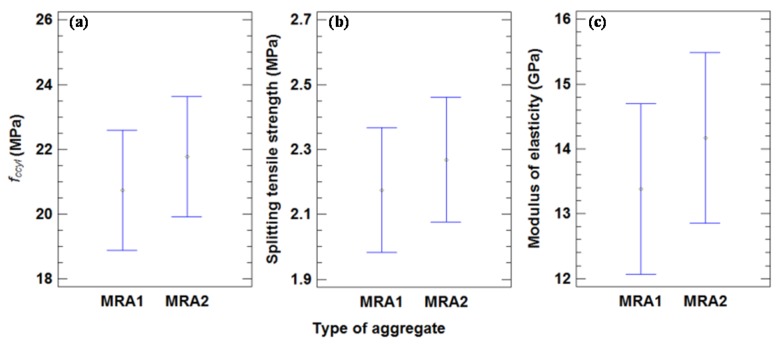
(**a**) Mean values of compressive strength in cylindrical specimens and 95% LSD intervals *vs.* type of MRA. (**b**) Mean values of splitting tensile strength compressive strength in cylindrical specimens and 95% LSD intervals *vs.* type of MRA. (**c**) Mean values of modulus of elasticity and 95% LSD intervals *vs.* type of MRA.

There were no statistically significant differences between the mechanical properties analyzed and the type of aggregate, as shown by the overlapping bars in Figure 12. This statistical result only confirms that the RA used in this work had similar characteristics. As a comparison between the two types of aggregates, the MRA1 mean compressive strength at 28 days was 20.74 MPa, and the same value for MRA2 was 21.85 MPa. This may be because the Los Angeles value of MRA1 is higher than that of MRA2, as observed by Ramesh Kumar and Sharma [54].

**Figure 13:**
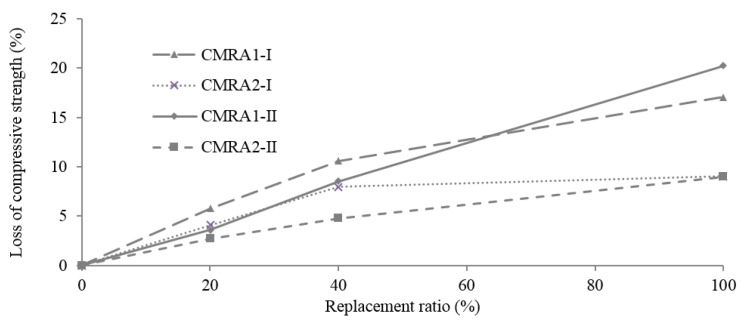
Loss of compressive strength, *f*_ccyl_, relative to CC with the replacement ratio.

Although there is no significant difference in the magnitude of the compressive strength between each type of aggregate, the results suggest a clear trend that does differ by the two types studied (Figure 13). The reduction of the compressive strength between the CMRA1-100-I/II and CC-I/II (9% for both series I and II) is greater than that of CMRA2-100-I/II (17.1% for series I and 20.2% for series II). MRA2 is apparently less harmful to concrete mixtures than MRA1.

The mean splitting tensile strength after 28 days for each type of aggregate was very similar: 2.17 and 2.27 MPa for MRA1 and MRA2, respectively. The higher strength of MRA2 may be due to the minor Los Angeles coefficient of this aggregate, which occurs for the compressive strength. The difference in splitting tensile strength was 4.3%, which is similar to the difference in compressive strength (5.0%).

The mean value of the modulus of elasticity for the concrete mixtures with MRA1 was 13.38 GPa, which was 5.6% lower than the mean value with MRA2 (14.17 GPa). These results were similar to the compressive strength decrease (5.0%). However, there was no significant difference between the modulus of elasticity and two types of aggregates, although the results show a greater reduction in the modulus of elasticity for CMRA1-100-I/II in relation to CC-I/II (31.5% for I and 33.9% for II) than CMRA2-100-I/II to CC-I/II (22.3% for I and 25.7% for II) due to the minor Los Angeles coefficient of this aggregate.

#### 4.3.2. Physical Properties

**Figure 14:**
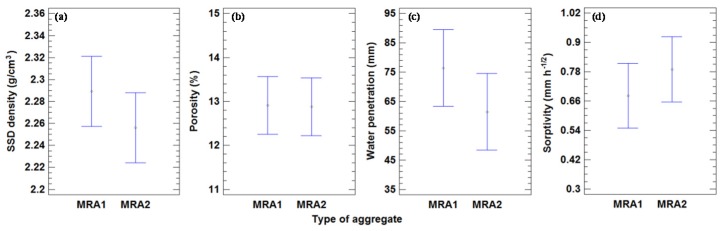
(**a**) Mean values of SSD density and 95% LSD intervals *vs.* type of MRA. (**b**) Mean values of porosity and 95% LSD intervals *vs.* type of MRA. (**c**) Mean values of water penetration and 95% LSD intervals *vs.* type of MRA. (**d**) Mean values of sorptivity and 95% LSD intervals *vs.* type of MRA.

There were no significant differences between the physical properties analyzed and the type of aggregate, as seen in Figure 14. The mean SS densities for MRA1 and MRA2 were 2.29 and 2.26 Mg/m^3^, respectively. The low density of concretes with MRA2 was due to the low density of this aggregate, as seen in Table 1.

The mean values of maximum water penetration for MRA1 and MRA2 were 76.4 and 62.1 mm, respectively, with a decline of 18.7%. These great penetration values exert an appreciable influence on the durability of concrete. Nevertheless, this result is not restrictive for the main purpose of this work, which is to manufacture a non-structural concrete to use without steel bars for reinforcement.

Mean sorptivity for MRA1 and MRA2 was 0.68 and 0.79 mm·h^−1/2^, respectively. These results agreed with those of Etxeberría *et al.* [27], who obtained mean sorptivity values of 0.515 mm·h^−1/2^ for MRA with cement content of between 240 and 265 kg/m^3^.

## 5. Conclusions

The mechanical and physical properties of concrete made with MRA and low cement content were analyzed in manufacturing a non-structural, low-strength concrete (15 MPa) using the highest substitution percentage of MRA in the coarse fraction. Based on the results obtained in this study, the following conclusions can be drawn:

- The main factors that affect the properties analyzed in this research are the amount of cement and the replacement ratio.
- The type of aggregate used in this research had no statistically significant effects on the properties analyzed.
- Excellent linear correlations between the percentage of substitution and loss of compressive strength, tensile strength, and the modulus of elasticity were found. These losses decrease with the amount of cement.
- Excellent linear correlations between the replacement ratio and increases in porosity, depth of water penetration under pressure and sorptivity were found. These are properties that adversely affect the durability, but the correlations do not present a negative impact for the purpose of this study, as a concrete without steel reinforcement is being sought.
- A replacement ratio of up to 20% of coarse natural aggregates by MRA presents no statistically significant differences in strength properties compared with the control concrete.
- It is possible to achieve a non-structural, low-strength concrete (15 MPa) with an MRA replacement ratio of up to 100% with 200 kg/m^3^ of cement. Previous studies have used cement quantities exceeding 240 kg/m^3^ for manufacturing non-structural concretes with MRA.
- Even though non-structural concrete made with MRA is not allowed by Spanish Code EHE-08, the results obtained here support its viability. Experimentation on a larger scale is required to confirm these results. This concrete could be used in the construction of ditches, sidewalks, and similar works, with the environmental benefits indicated above.
